# Association between radiotherapy for surgically treated oral cavity cancer and secondary lung cancer

**DOI:** 10.3389/fpubh.2023.1120671

**Published:** 2023-03-22

**Authors:** Dongjie He, Jun Zhang, Ying Xiang, Peiwen Wu, Gaiyan Li, Hao Chang, Qiming Wang, Qiuju Shao, Siying Zhu

**Affiliations:** ^1^Department of Radiation Oncology, Tangdu Hospital, The Second Affiliated Hospital of Air Force Military Medical University, Xi'an, China; ^2^Department of Otolaryngology, Tangdu Hospital, The Second Affiliated Hospital of Air Force Military Medical University, Xi'an, China; ^3^Department of Traditional Chinese Medicine, The Sixth Medical Center of PLA General Hospital, Beijing, China

**Keywords:** oral cavity cancer, radiotherapy, secondary lung cancer, SEER, survivors

## Abstract

**Background:**

There is limited research on the incidence of secondary lung cancer (SLC) after radiotherapy (RT) for oral cavity cancer (OCC). Therefore, we investigated the association between RT for OCC and the risk of SLC and the overall survival of these patients.

**Methods:**

Patients diagnosed with OCC between 1975 and 2015 were selected from the Surveillance, Epidemiology, and End Results database. The cumulative incidence of SLC, relative risk (RR) of RT vs. no RT (NRT), standardized incidence ratios (SIR), and survival outcomes were assessed.

**Results:**

A total of 10,936 patients with OCC were included. Of these, 429 (3.92%) patients developed SLC, where 136 (5.02%) received RT and 293 (3.56%) did not. The cumulative incidence of SLC during follow-up was 6.89% and 4.84% in the RT and NRT patients, respectively. RT was associated with a higher risk of SLC. In the subset analysis, the results showed that a higher risk of developing SLC among patients with index OCC in most subgroups. Dynamic RR and SIR revealed a decreased risk of SLC with increasing latency time. No difference was observed in the 10-year survival rates for patients with SLC who received RT or not or compared with primary lung cancer.

**Conclusion:**

RT was associated with a higher risk of SLC, and patients diagnosed with OCC could be followed for 5–10 years after diagnosis.

## Summary

This study aimed to investigate the association between radiation given as treatment for surgically treated oral cavity cancer and the occurrence of lung cancer as secondary cancer. Here, we show that radiation has a greater risk of causing secondary lung cancer, particularly 5–10 years after the oral cancer diagnosis. In addition, these findings support extended follow-up time in patients who have received radiation for oral cavity cancer.

## 1. Introduction

Oral cavity cancer (OCC) is one of the most common malignancies of the head and neck and the sixth most common cancer worldwide (1). In the United States, the annual incidence of OCC is estimated to be between 4 and 4.3 cases per 100,000, and the burden of OCC cases is steadily increasing (2). OCC also represents a growing health concern since, in 2018, it was estimated to have a death toll of 119,700 men and 57,700 women worldwide (3). However, the number of survivors of OCC has been increasing, indicating that cancer treatment is advancing (4). Unfortunately, these survivors experience an elevated risk of developing a second primary cancer (5–8), which is the leading cause of mortality in these patients (9).

Currently, the standard treatment for early-stage resected OCC with high-risk features is adjuvant radiochemotherapy, and the strategy for advanced OCC is surgical resection, adjuvant radiotherapy (RT), and chemotherapy (10, 11). However, the adverse effects caused by RT for OCC are significant, including severe disruption of the patient's quality of life and long-term side effects, such as radiation injury and second primary cancers (12, 13). In addition, lung cancer is becoming increasingly common as a second primary malignancy. Several retrospective studies have reported a phenomenon that RT was associated with an increased risk of developing secondary lung cancer (SLC) following head and neck cancer (14, 15). Similarly, RT for some pelvic cancer has also been reported to increase the risk of SLC (16–18). However, there is limited research on the association between RT and SLC incidence in patients with OCC.

Therefore, this retrospective study aimed to evaluate the association between RT for OCC and the incidence of SLC, as well as the survival rate of SLC after RT in patients with OCC.

## 2. Materials and methods

### 2.1. Patient selection

The Surveillance, Epidemiology, and End Results (SEER) database, which includes nine population-based registries from the USA between 1975 and 2015, was used for patient se-lection and data collection (7). Patients meeting the following criteria were included: a diagnosis of OCC (site codes C003-009, C020-023, C030-039, C040-050, C053-059, and C060-069) (19, 20); primary cancer; and an accurate record of surgical interventions. The exclusion criteria were as follows: death certificate or autopsy records, multiple cancers, age <20 years, distant and unknown stage at diagnosis according to the SEER Combined Summary Stage and Historic Stage A variables, unknown or no surgical information, unknown modality of radiation therapy, and survival of <60 months after oral cavity diagnosis (7). Supplementary Figure S1 shows a flow diagram of the inclusion/exclusion criteria and the study design.

The following data were collected from the SEER database for each patient: age, sex, race, marital status, anatomic sites, histology, grade, stage, chemotherapy, and follow-up time. Patients with OCC were categorized into the following two groups: RT and no RT (NRT). Informed patient consent was not required to access or use the SEER data. Therefore, the requirement for ethical approval and informed consent was waived for the present study. This study followed the Strengthening the Reporting of Observational Studies in Epidemiology (STROBE) reporting guidelines.

### 2.2. Definition of SLC and follow-up

Given the complexity of SLC diagnosis and the high likelihood of incident malignancy detection immediately after OCC diagnosis, follow-up for the analysis of SLC was initiated 60 months following the diagnosis of OCC, considering the minimum latency for radiation-induced cancerization (21). Therefore, the latency period for SLC began 5 years after OCC diagnosis and ended at the date of diagnosis of any SLC, all-cause death, or after 30 years of follow-up, whichever occurred first (7, 22).

### 2.3. Statistical analyses

Fine-Gray models were used to assess the cumulative incidence and risks of SLC after the diagnosis of OCC (7). The occurrence of non-SLC and all-cause mortality were considered competing events. These factors were considered in calculating hazard ratios (HRs) and 95% confidence intervals (CIs) for developing SLC (22). Multivariable competitive regression and Poisson regression were used to assess the risk of SLC development. Subgroup analysis was performed using a competing risk regression model. Relevant clinical interventions were included in the model.

Poisson regression was used to compare the RT-relative risk (RR) and 95% CI of developing SLC in patients with OCC who received RT and NRT. In addition, the standardized incidence ratio (SIR) and 95% CI were estimated using a Poisson regression analysis. SIR was estimated using SEER^*^Stat 8.4.0.1. In the RR and SIR adjustment model, we included the following three variables in our retrospective study: age at OCC diagnosis, year of OCC diagnosis, and latency time of SLC diagnosis. To further evaluate the dynamic risks and incidence of SLC associated with RT, we calculated RR and SIR stratified by latency time since OCC diagnosis, age at OCC diagnosis, and year of OCC diagnosis.

Propensity-score matching was used to reduce bias. A one-to-one nearest neighbor matching algorithm was used (23, 24). We matched the baseline characteristics of SLC, including age, sex, race, years of diagnosis, marital status, sites, grade, histology, stage, surgery, radiation, and chemotherapy, to assess survival in the RT vs. NRT group for SLC. The 10-year overall survival (OS) of SLC between RT and NRT groups was compared using Kaplan–Meier curves before and after propensity-score matching. To compare the 10-year OS between PLC and SLC after OCC, we also matched SLC according to their baseline characteristics, including age, sex, race, years of diagnosis, marital status, sites, grade, histology, stage, surgery, radiation, and chemotherapy. Differences in OS were compared using the log-rank test. All analyses were performed using R Statistical Software 4.1.1 (http://www.R-project.org, The R Foundation) and Free Statistics software version 1.7.0 (Based on R statistical software 3.2.2) (24, 25). A *p*-value <0.05 was considered statistically significant.

## 3. Results

### 3.1. Baseline characteristics of patients with OCC

A total of 10,936 patients who were diagnosed with OCC and underwent surgery were identified according to the eligibility criteria, and their baseline characteristics are shown in Table 1. In addition, 29.9% of adult patients with tumors at the localized and regional stages survived for more than 5 years. Of these, 24.8% and 75.2% of the patients were treated with and without RT, respectively. The median age at diagnosis was 60 years, the median follow-up time was 140 months, and the median latency time was 113 months. The RT group was younger (58 years), more often male (*n* = 1,626, 60.0%), less often white (*n* = 2,266, 83.6%), more often diagnosed in later years, less often had grade I/II OCC (*n* = 1,805, 66.6%), more often squamous cell carcinoma (*n* = 2,293, 84.6%), less often localized staging (*n* = 938,34.6%), and more often received chemotherapy (*n* = 413, 15.2%), compared with the NRT group. Of the 10,936 identified cases, 429 (3.92%) developed SLC during the follow-up period, with 136 (5.02%) and 293 (3.56%) in the RT and NRT groups, respectively.

**Table 1:** Baseline characteristics of patients with surgically treated OCC.

**Variables**	**Total**	**Radiotherapy**	**No radiotherapy**	***p*-value**
Number, *n* (%)	10,936 (100.0)	2,710 (24.8)	8,226 (75.2)	
Age, median (IQR), year	60.0 (51.0, 69.0)	58.0 (51.0, 67.0)	60.0 (51.0, 69.0)	<0.001
**Age**, ***n*** **(%), year**				<0.001
20–49	2,418 (22.1)	602 (22.2)	1,816 (22.1)	
50–69	5,983 (54.7)	1,586 (58.5)	4,397 (53.5)	
≥70	2,535 (23.2)	522 (19.3)	2,013 (24.4)	
**Sex**, ***n*** **(%)**				0.003
Female	4,639 (42.4)	1,084 (40.0)	3,555 (43.2)	
Male	6,297 (57.6)	1,626 (60.0)	4,671 (56.8)	
Race, *n* (%)				<0.001
White	9,505 (86.9)	2,266 (83.6)	7,239 (88.0)	
Black	686 (6.3)	236 (8.7)	450 (5.5)	
Other/unknown^a^	745 (6.8)	208 (7.7)	537 (6.5)	
Year, median (IQR)	1,996 (1,986, 2,005)	1,997 (1,987, 2,006)	1,995 (1,985, 2,005)	<0.001
**Year**, ***n*** **(%)**				<0.001
1975–1984	2,429 (22.3)	518 (19.1)	1,911 (23.2)	
1985–1994	2,738 (25.0)	666 (24.6)	2,072 (25.2)	
1995–2004	2,878 (26.3)	751 (27.7)	2,127 (25.9)	
≥2005	2,891 (26.4)	775 (28.6)	2,116 (25.7)	
**Marital status**, ***n*** **(%)**				0.086
Single	1,476 (13.5)	397 (14.6)	1,079 (13.1)	
Married	6,406 (58.6)	1,584 (58.5)	4,822 (58.6)	
Other/unknown^b^	3,054 (27.9)	729 (26.9)	2,325 (28.3)	
**Anatomic sites**, ***n*** **(%)**				<0.001
Lip	919 (8.3)	41 (1.5)	878 (10.8)	
Tongue	3,285 (30.0)	778 (28.7)	2,507 (30.5)	
Gum	1,188 (10.9)	346 (12.8)	842 (10.2)	
Floor of mouth	2,775 (25.4)	773 (28.5)	2,002 (24.3)	
Palate	947 (8.7)	196 (7.2)	751 (9.1)	
Other	1,822 (16.7)	576 (21.3)	1,246 (15.1)	
**Grade**, ***n*** **(%)**				<0.001
Grade I/II	7,496 (68.5)	1,805 (66.6)	5,691 (69.2)	
Grade III/IV	995 (9.1)	448 (16.5)	547 (6.6)	
Unknown	2,445 (22.4)	457 (16.9)	1,988 (24.2)	
**Histology**, ***n*** **(%)**				0.409
Squamous cell carcinoma	8,832 (80.8)	2,293 (84.6)	6,539 (79.5)	
Other	2,104 (19.2)	417 (15.4)	1,687 (20.5)	
Stage, *n* (%)				<0.001
Localized	7,361 (67.3)	938 (34.6)	6,423 (78.1)	
Regional	3,575 (32.7)	1,772 (65.4)	1,803 (21.9)	
**Chemotherapy**, ***n*** **(%)**				<0.001
No	10,473 (95.8)	2,297 (84.8)	8,176 (99.4)	
Yes	463 (4.2)	413 (15.2)	50 (0.6)	
Follow-up time, median (IQR), months	140.0 (94.0, 211.0)	125.0 (88.0, 191.0)	145.0 (97.0, 218.0)	<0.001
Secondary lung cancer, *n* (%)	429 (3.92)	136 (5.02)	293 (3.56)	<0.001
Latency time, median (IQR), months	113 (81, 165)	105 (77, 138)	119 (82, 170)	0.016

IQR, interquartile range; OCC, oral cavity cancer.

a Other including American Indian/AK Native, Asian/Pacific Islander.

b Other including Divorced, Separated, Widowed, Unmarried or Domestic partner.

### 3.2. Cumulative incidence of SLC

**Figure 1:**
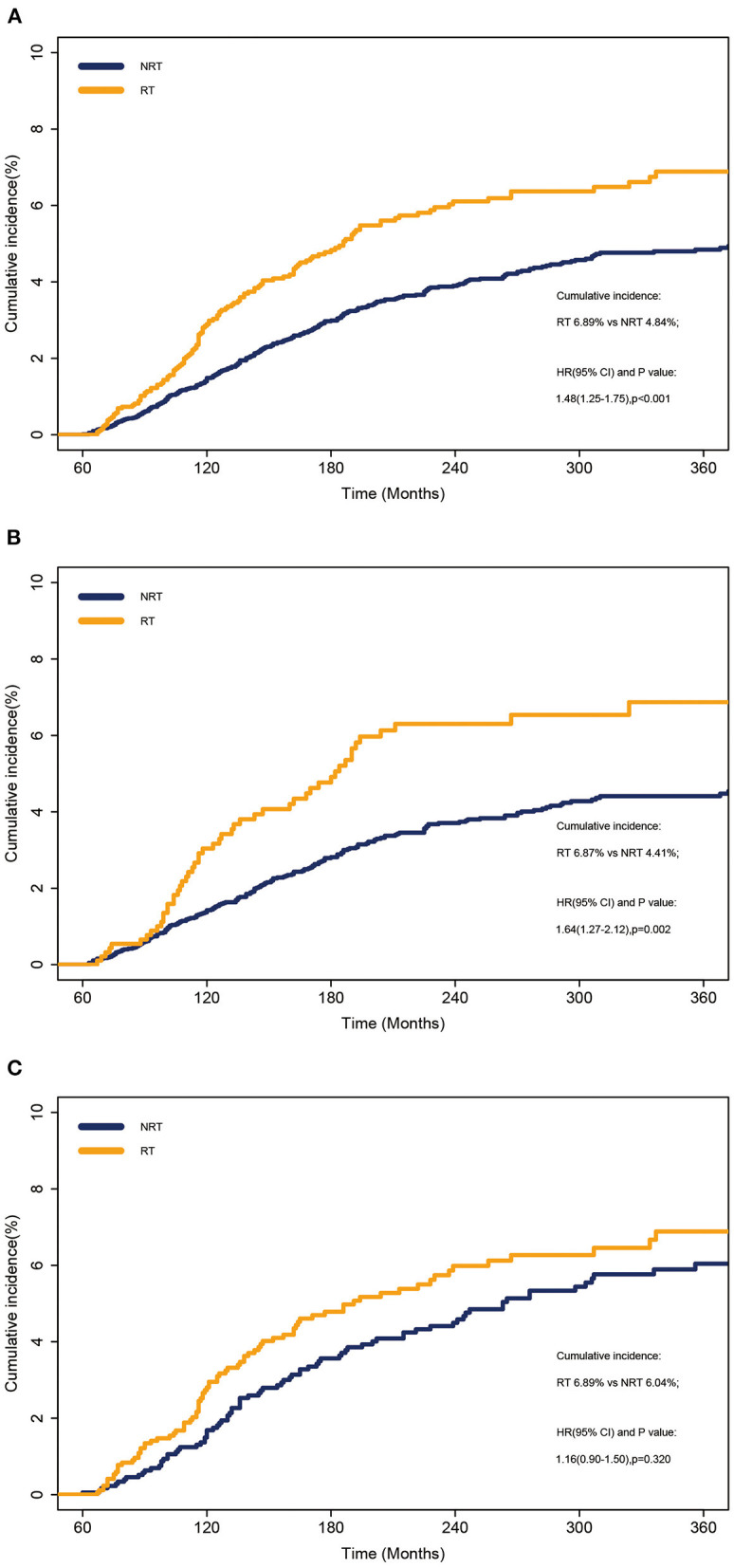
Cumulative incidence of SLC between patients who received RT and those who did not received RT. **(A)** Overall cohort. **(B)** Localized disease. **(C)** Regional disease. NRT, no radiotherapy; RT, radiotherapy.

The cumulative incidence of SLC was calculated at 6.89% and 4.84% in the RT and NRT groups, respectively, using a Fine-Gray model (Figure 1A). The incidence of SLC in the RT group was higher than that in the NRT group (*p* < 0.001) (Figure 1A). Similarly, patients showed a significant increase in mortality with RT compared to NRT (80.60% vs. 76.54%; *p* < 0.001; Supplementary Figure S2). Furthermore, we performed a stratified analysis on the occurrence of SLC by stage. The incidence of SLC in the RT group was higher than that in the NRT group for localized disease (HR = 1.64, 95% CI: 1.27–2.12; *p* = 0.002) (Figure 1B). The incidence of SLC was not different in the RT and NRT groups for regional disease (HR =1.16, 95% CI: 0.90–1.50; *p* = 0.320) (Figure 1C). As shown in Table 2, the multivariate competing risk model revealed that a radiation history of patients with OCC was an independent risk factor for SLC (HR = 1.35, 95% CI: 1.11–1.65; *p* = 0.010). Poisson regression was used to assess the relative risk of SLC without considering competing events. Multivariate Poisson regression analyses showed that patients with OCC who received RT significantly increased the RR of SLC vs. those with NRT (RR = 1.33, 95% CI: 1.09–1.60; *p* = 0.015; Supplementary Table S1).

**Table 2:** Competing risk regression model for developing SLC in OCC.

**Variables**	**Univariate analysis**		**Multivariate analysis**	
	**HR (95% CI)**	***p***	**HR (95% CI)**	***p***
**Age**				
20–49	1 (Ref)		1 (Ref)	
50–69	2.25 (1.78–2.85)	<0.001	1.85 (1.45–2.36)	<0.001
≥70	1.01 (0.75–1.37)	0.950	0.98 (0.72–1.34)	0.910
**Sex**				
Female	1 (Ref)		1 (Ref)	
Male	1.45 (1.23–1.72)	<0.001	1.28 (1.07–1.53)	0.021
**Race**				
White	1 (Ref)		1 (Ref)	
Black	1.14 (0.85–1.55)	0.460	1.07 (0.79–1.46)	0.700
Other^a^	0.57 (0.37–0.88)	0.032	0.71 (0.46–1.10)	0.200
**Year**				
1975–1984	1 (Ref)		1 (Ref)	
1985–1994	0.88 (0.73-1.07)	0.280	0.98 (0.81–1.19)	0.870
1995–2004	0.68 (0.55-0.83)	0.002	0.81 (0.65–1.01)	0.110
≥2005	0.40 (0.28-0.56)	<0.001	0.47 (0.33–0.67)	<0.001
**Marital status**				
Single	1 (Ref)		1 (Ref)	
Married	1.30 (0.99–1.71)	0.120	1.21 (0.92–1.60)	0.250
Other/unknown^b^	1.39 (1.04–1.86)	0.065	1.37 (1.01–1.85)	0.085
**Site**				
Lip	1 (Ref)		1 (Ref)	
Tongue	0.81 (0.57–1.14)	0.310	0.80 (0.56–1.15)	0.320
Gum	0.93 (0.62–1.40)	0.790	0.97 (0.64–1.49)	0.920
Floor of mouth	1.97 (1.43–2.72)	<0.001	1.62 (1.16–2.26)	0.019
Palate	0.57 (0.35–0.93)	0.060	0.94 (0.56–1.58)	0.850
Other	1.34 (0.94–1.89)	0.170	1.41 (0.98–2.04)	0.120
**Grade**				
Grade I/II	1 (Ref)		1 (Ref)	
Grade III/IV	1.13 (0.86–1.48)	0.470	1.02 (0.77–1.34)	0.930
Unknown	0.99 (0.82–1.20)	0.960	1.07 (0.88–1.30)	0.570
**Histology**				
Squamous cell carcinoma	1 (Ref)		1 (Ref)	
Other	0.44 (0.34–0.58)	<0.001	0.53 (0.39–0.72)	0.001
**Stage**				
Localized	1 (Ref)		1 (Ref)	
Regional	1.34 (1.14–1.58)	0.003	1.01 (0.83–1.22)	0.960
**Chemotherapy**				
No	1 (Ref)		1 (Ref)	
Yes	1.37 (0.92–2.04)	0.190	1.16 (0.76–1.75)	0.560
**Radiotherapy**				
No	1 (Ref)		1 (Ref)	
Yes	1.48 (1.25–1.75)	<0.001	1.35 (1.11–1.65)	0.010

SLC, second primary lung cancer; OCC, oral cavity cancer; HR, hazard ratio; CI, confidence interval, Ref., Reference.

a Other including American Indian/AK Native, Asian/Pacific Islander.

b Other including Divorced, Separated, Widowed, Unmarried or Domestic partner.

**Figure 2:**
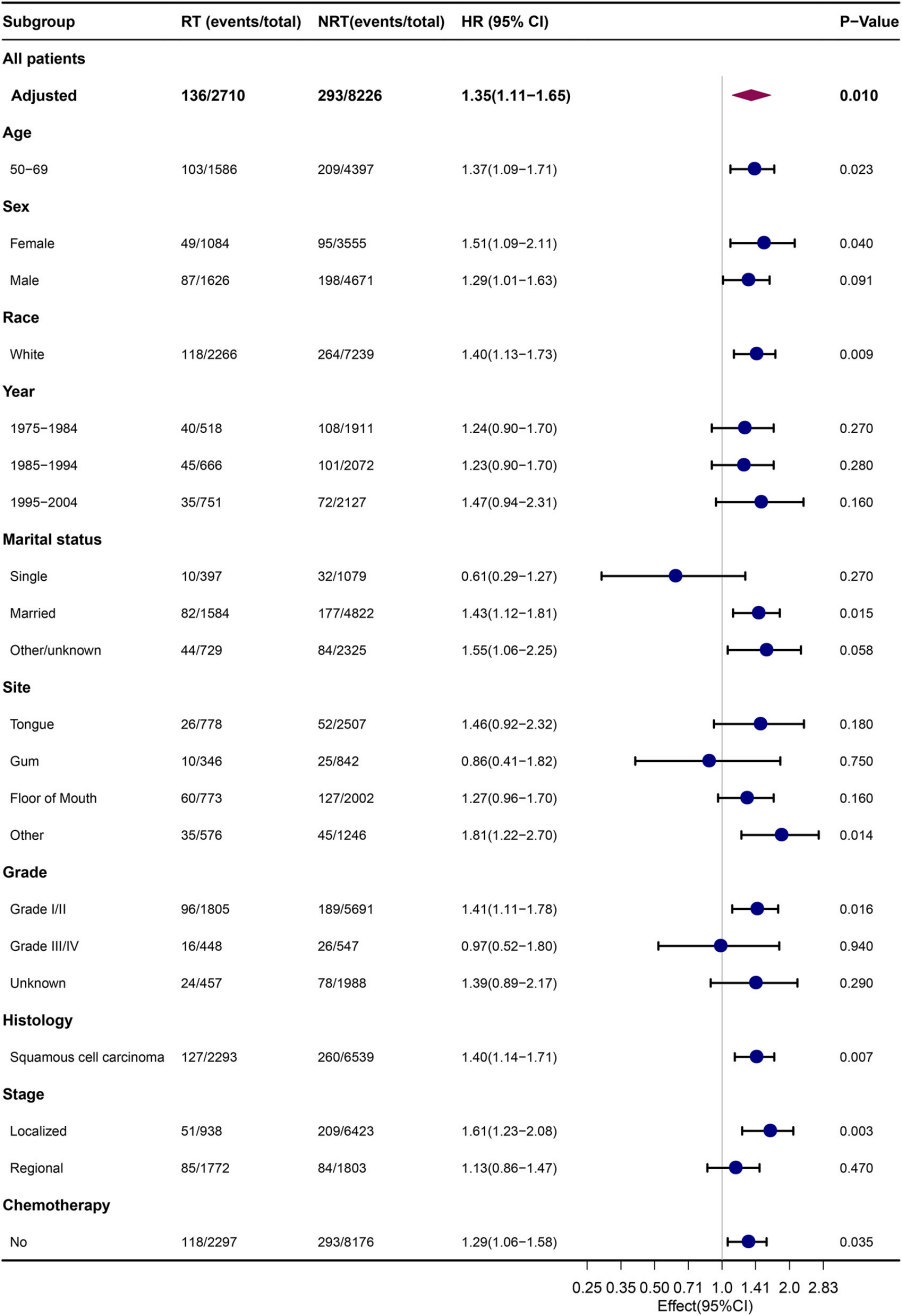
Comparison of risk of SLC between subgroups by estimating HRs through competing risk analyses. NRT, no radiotherapy; RT, radiotherapy; HRs, hazard ratios.

Subgroup analyses were performed to compare the risk of developing SLC in patients with OCC with or without previous RT. The results of the competing risk analysis (Figure 2; Supplementary Table S2) showed a higher risk of developing SLC among patients with OCC who were aged between 50 and 69 years (HR = 1.37, 95% CI: 1.09–1.71), female (HR = 1.51, 95% CI: 1.09–2.11), white (HR = 1.40, 95% CI: 1.13–1.73), married (HR = 1.43, 95% CI: 1.12–1.81), diagnosed with squamous cell carcinoma (HR = 1.40, 95% CI: 1.14–1.71), diagnosed with localized diseases (HR = 1.61, 95% CI: 1.23–2.08) or received no chemotherapy (HR = 1.29, 95% CI: 1.06–1.58).

### 3.3. Dynamic risk and incidence evaluation for SLC

**Figure 3:**
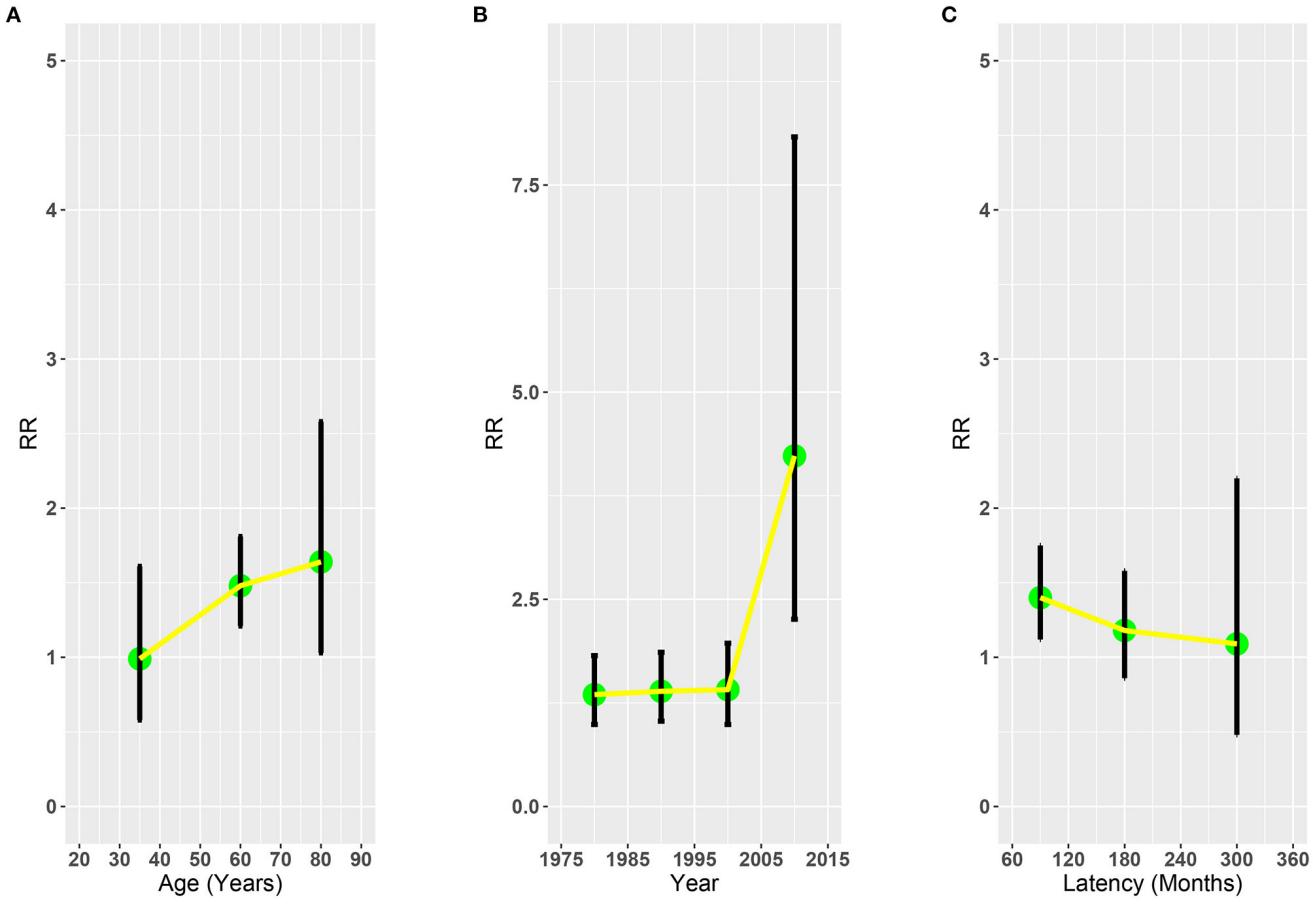
Adjusted relative risk plots of SLC of patients with OCC treated with RT or without RT. **(A)** Age at OCC diagnosis. **(B)** Year of OCC diagnosis. **(C)** Latency time. RR, relative risk; OCC, oral cavity cancer; RT, radiotherapy; SLC, second primary lung cancer.

To further evaluate the risk of SLC associated with RT, we analyzed the RR plot of the latency time after OCC diagnosis, diagnosis year of OCC, and age at primary OCC diagnosis. The results of the multivariate Poisson regression revealed that RT was associated with elevated risks of SLC in patients aged 50–69 years with OCC (RR = 1.48, 95% CI: 1.21–1.81, *p* = 0.001; Figure 3A). No significant changes were observed in other age groups (20–49: RR = 0.99, 95% CI: 0.58–1.61, *p* = 0.978; ≥70: RR = 1.64, 95% CI: 1.03–2.58, *p* = 0.073; Figure 3A). In the RR plots for the years since diagnosis, we found that the risk increased and reached a maximum between 2005 and 2015 (1975–1984: RR = 1.35, 95% CI: 0.99–1.82; 1985–1994: RR = 1.39, 95% CI 1.03–1.86; 1995–2004: RR = 1.41, 95% CI: 0.99–1.97; ≥2005: RR = 4.23, 95% CI 2.26–8.08; Figure 3B). However, in the RR plot of latency time, we found that the risk of SLC decreased with the prolongation of latency time of OCC diagnosis (60–119: RR = 1.40, 95% CI: 1.12–1.75; 120–239: RR = 1.18, 95% CI: 0.86–1.58; 240–360: RR = 1.09, 95% CI: 0.48–2.20; Figure 3C).

In addition, we evaluated the SIR of SLC in the US population in patients with OCC with or without a prior history of RT, grouped by the latency time after OCC diagnosis, year of primary OCC diagnosis, and age at primary OCC diagnosis. We found that the patients with OCC who received RT (SIR: 3.07, 95% CI: 2.78–3.38, *p* < 0.05) and those who did not (SIR: 2.41, 95% CI: 2.22–2.61, *p* < 0.05) had a higher risk of SLC than the general population (Supplementary Figure S3).

### 3.4. Survival outcome of SLC

**Figure 4:**
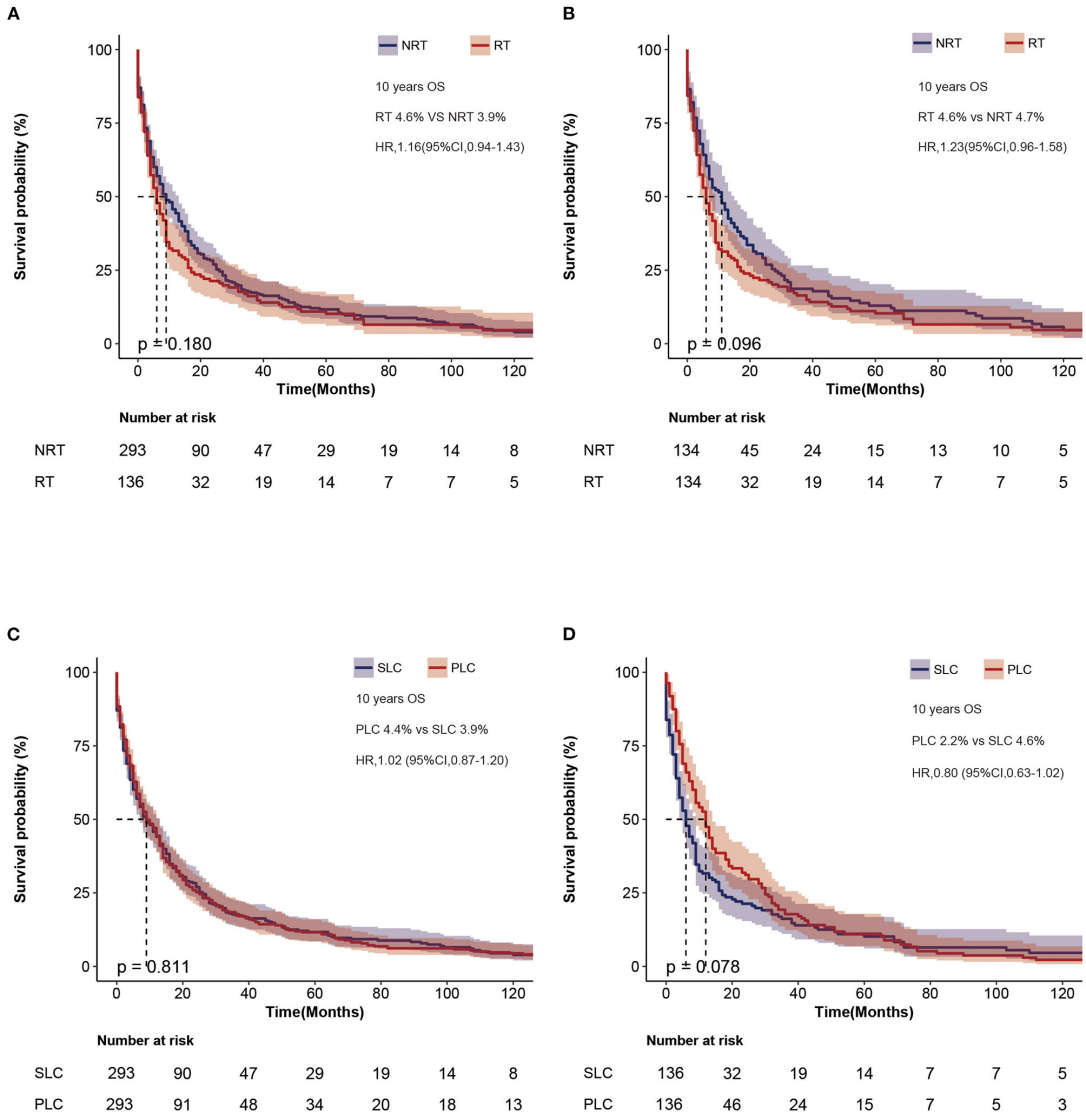
Survival outcome of patients with SLC. **(A)** Overall survival after diagnosis of second lung cancer before matching. **(B)** Overall survival of second lung cancer between RT and NRT groups after matching. **(C)** Overall survival between PLC and SLC after matching in the NRT group. **(D)** Overall survival between PLC and SLC after matching in the RT group. SLC, second primary lung cancer.

We compared the OS of patients with SLC who received RT with the NRT group, and the 10-year survival rates were not significantly different (Figure 4A). Therefore, to reduce bias, we performed a propensity score (Supplementary Table S3), and the 10-year survival rates were 4.6% and 4.7% for the RT and NRT groups, respectively. However, no significant difference was observed in the 10-year survival (HR = 1.23, *p* = 0.096) (Figure 4B). To further analyze the survival of SLC, we matched SLC to primary lung cancer (PLC) separately (Supplementary Table S4, Supplementary Figure S1). The 10-year survival rates of SLC and PLC were 3.9% and 4.4% in the NRT group, and 4.6% and 2.2% in the RT group. In addition, no difference was found in survival between SLC and PLC (PLC vs. SLC, HR = 1.02, 95% CI: 0.87–1.20, *p* = 0.811, Figure 4C; PLC vs. SLC, HR = 0.80, 95% CI: 0.63–1.02, *p* = 0.078, Figure 4D).

## 4. Discussion

RT effectively improves OS and local progression-free survival in patients with OCC (10, 26). However, it remains unclear whether RT is associated with SLC. Therefore, we investigated this association by selecting patients with OCC from the SEER database and comparing those with and without a history of RT. First, our findings show that, RT was associated with an increased risk of SLC in patients with OCC. Second, in the US population, the risk of patients with SLC who received RT was higher than that of those who did not receive RT. Third, the risk of developing SLC after RT in patients with OCC increased with increasing age at diagnosis and year of diagnosis and decreased with increasing latency between OCC and SLC diagnoses. Fourth, no significant difference was observed in the survival of patients with SLC who had or had not received RT and that of those with SLC or with PLC.

Previous studies have shown that the risk of developing lung cancer in patients with laryngeal cancer treated with RT (7, 14, 27). In addition, the study showed that RT of the first primary tumor might result in developing secondary solid tumors in 8% of patients (28). However, evidence from several studies has shown that RT is not associated with an increase in the diagnosis of second primary cancers (29, 30). Therefore, developing secondary tumors after RT for head and neck tumors remains controversial, and there are few studies on SLC after RT for OCC. Previous studies have reported that most head and neck tumors develop distant metastases within 2 years (31), and radiotherapy-induced tumors develop within more than 5 years (7). To remove these confounding factors, we included patients who survived for at least 5 years and had an interval of 5 years or more between the two tumors. Our study showed that the patients who received RT had a higher risk of developing SLC than those who did not. We further performed a stratified analysis by stage and found that the patients with localized disease had a higher risk of SLC in the RT group than in the NRT group. Simultaneously, no difference was found between the two in the regional disease. The following subgroup analysis of the multivariate competing risk model also reflects this result. A possible explanation is that patients with localized stage have better survival and are more likely to develop SLC (32, 33). To better illustrate these results, we used both the competitive risk model (HR = 1.35, 95% CI: 1.11–1.65; *p* = 0.010) and the Poisson regression model (RR = 1.33, 95% CI: 1.09–1.60; *p* = 0.015) to obtain similar results. The subgroup analysis revealed that prior RT history exhibited a higher risk of developing SLC for patients who were aged 50–69 years, white, diagnosed with squamous cell carcinoma, diagnosed with localized diseases, or had not received chemotherapy. These results are consistent with previous reports showing an increased risk of SLC secondary to RT in patients with nasopharyngeal carcinoma (34).

In this study, we dynamically evaluated the relationship between RT and SLC according to age, year of OCC, and latency to SLC. The results showed that a higher risk of SLC in the RT group was found between the ages of 50 and 69 years, there. This phenomenon can also be observed in PLC survivors (21). It is possible that a history of smoking may increase the risk of SLC in patients who are between the ages of 50 and 69 years. Therefore, the potential mechanism should be explored. We also showed that the risk of SLC increased with more recent years of diagnosis. The most likely explanation for this is that newer RT techniques have increased the delivery time of treatments, thus increasing the exposure time to normal tissue (35, 36). Our results are inconsistent with previous reports showing no difference in the risk of secondary tumors after the update in RT techniques. However, this may be due to the short duration of radiation exposure used in the inclusion criteria (37). We also evaluated the relationship between the time of primary OCC and SLC diagnosis and demonstrated that the risk was reduced with longer follow-up. Therefore, RT-associated SLC should be given more attention for 5–10 years after RT for OCC.

We also evaluated the risk of SLC associated with RT in the general population. The results suggest that the risk increased in the general population (NRT: SIR = 2.41, range 2.22–2.61; RT: SIR = 3.07, range 2.78–3.38). Previous study also reported that there is a difference in outcome comparisons between the incidence of such speciation in the general population (38). We believe that the cause of SLC may be related to a combination of genetics, smoking, and lifestyle factors (28, 39, 40). In addition, the risk of finding SLC in patients with previous HNSCC was 2.5 times higher than that in those without tumors (41). In particular, tumors that have undergone curative treatment are at a higher risk of developing SLC (42). With this study, we hope to raise awareness of the problem of SLC in patients with OCC.

Many factors, such as lifestyle habits, genetic mutations, and age, may contribute to SLC. Radiation-associated secondary tumors are related to the volume and dose of radiation to surrounding organs (43). Lung cancer is the most common of these radiation-associated solid tumors (15). One possible explanation is that low doses of radiation cause DNA damage, leading to malignant transformation (44, 45). Another possible explanation is the larger volume of irradiated normal tissue in the out-of-field or scattered radiation, leading to an increased risk of secondary tumor development (36). Therefore, there is a need to further investigate the mechanisms of SLC in patients surviving RT.

Propensity-score matching was performed to further explore RT-associated SLC. We found no difference in OS after SLC between the NRT and RT groups either before or after matching to SLC. However, the potential mechanism requires further investigation. In addition, we explored the OS of SLC vs. PLC and no differences were found between the two groups of patients. In addition, we explored the OS of SLC vs. PLC and found no differences between the two groups of patients. Similarly, recent data suggest that patients with SLC or PLC have a comparable OS (46, 47).

This study had some limitations. First, the SEER database does not record smoking status or duration, which is an important factor when considering lung cancer. Some studies have suggested an increased risk of SLC in patients with a heavy smoking history (39, 48). There may also be an interaction between tobacco and radiation in SLC (45). Conversely, other reports have shown that the risk of developing SLC was similar between smokers and never-smokers (49, 50). Without further investigations, conclusions cannot be drawn regarding the association between the presence of SLC and smoking. Therefore, we believe that this factor had a relatively small influence on our conclusions. Second, there are limited data in the SEER database on the indication, dose, and frequency of radiation therapy, specific information on chemotherapy, and molecular information on lung cancer. Third, RT was not randomized, and some retrospective biases may have affected the risk of SLC after RT vs. NRT. Fourth, it is challenging to distinguish PLC from distant metastasis since it is not always opportune to collect tumor tissue of the lung to be compared against the primary tumor. Our results demonstrate that RT may increase the risk of SLC in patients with oral cancer, but further studies are required to answer these questions.

## 5. Conclusion

RT for patients with primary OCC was associated with a higher risk of developing SLC than in those who were not exposed to RT. However, no significant difference was found in the survival of patients with OCC, SLC, and PLC. Nevertheless, we recommend 5–10 years of monitoring of the risk of SLC in patients with a history of RT for OCC.

## Data availability statement

Publicly available datasets were analyzed in this study. This data can be found here: www.seer.cancer.gov.

## Ethics statement

All data used was based on publicly available data in the SEER database. Ethical review and approval was therefore not required for the study on human participants in accordance with the local legislation and institutional requirements. The Ethics Committee waived the requirement of written informed consent for participation.

## Author contributions

Conceptualization and methodology: GL, JZ, SZ, and YX. Data analysis and writing—original draft preparation: DH and YX. Writing—review and editing: SZ and QS. Figure and table preparation: HC, PW, and QW. Supervision: GL and YX. All authors have read and agreed to the published version of the manuscript.

## Supplementary material

Supplementary Figure S1 Flowchart of the study cohort. PSM, Propensity Score Matching.

Supplementary Figure S2 Cumulative incidence and cumulative mortality of SLC between patients who received RT and those who did not receive RT.

Supplementary Figure S3 SIR for SLC by age at diagnosis, latency, and year of OCC diagnosis.

Supplementary Table S1 Poisson regression model for developing SLC in OCC.

Supplementary Table S2 Subgroup analyses of competing risk regression for the risk of developing SLC.

Supplementary Table S3 Baseline characteristics of patients with SLC in the original/matched data set.

Supplementary Table S4 Baseline characteristics of patients with SLC and matched PLC in OCC.
