# Supplementary figures and images for: Association between radiotherapy for surgically treated oral cavity cancer and secondary lung cancer
Source: Front Public Health. 2023 Mar 22;11:1120671. doi: 10.3389/fpubh.2023.1120671 (PMC10073750; doi:10.3389/fpubh.2023.1120671)

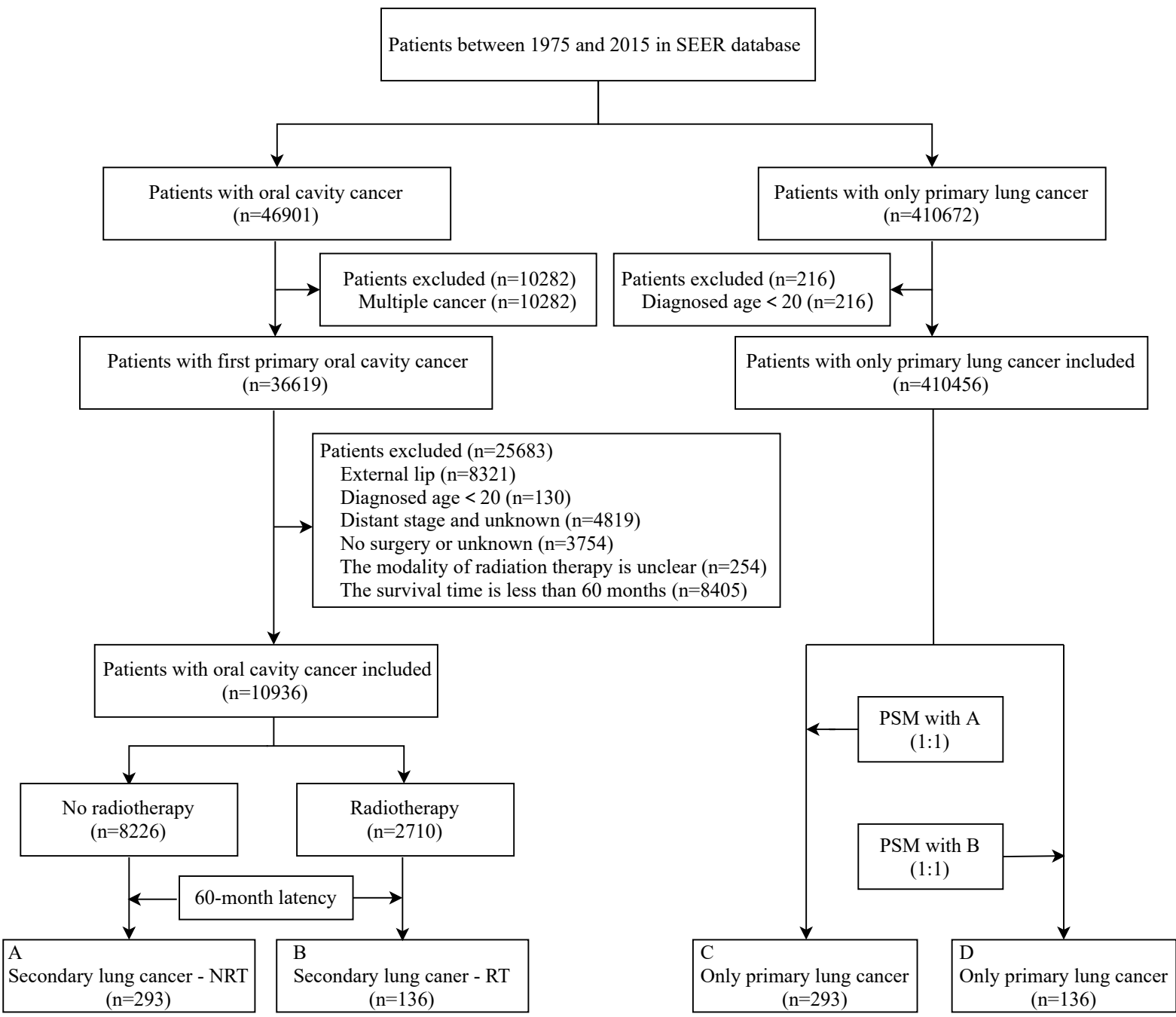

Supplement: Supplementary Figure S1 — Flowchart of the study cohort. PSM, Propensity Score Matching. [file Data_Sheet_1.PDF]

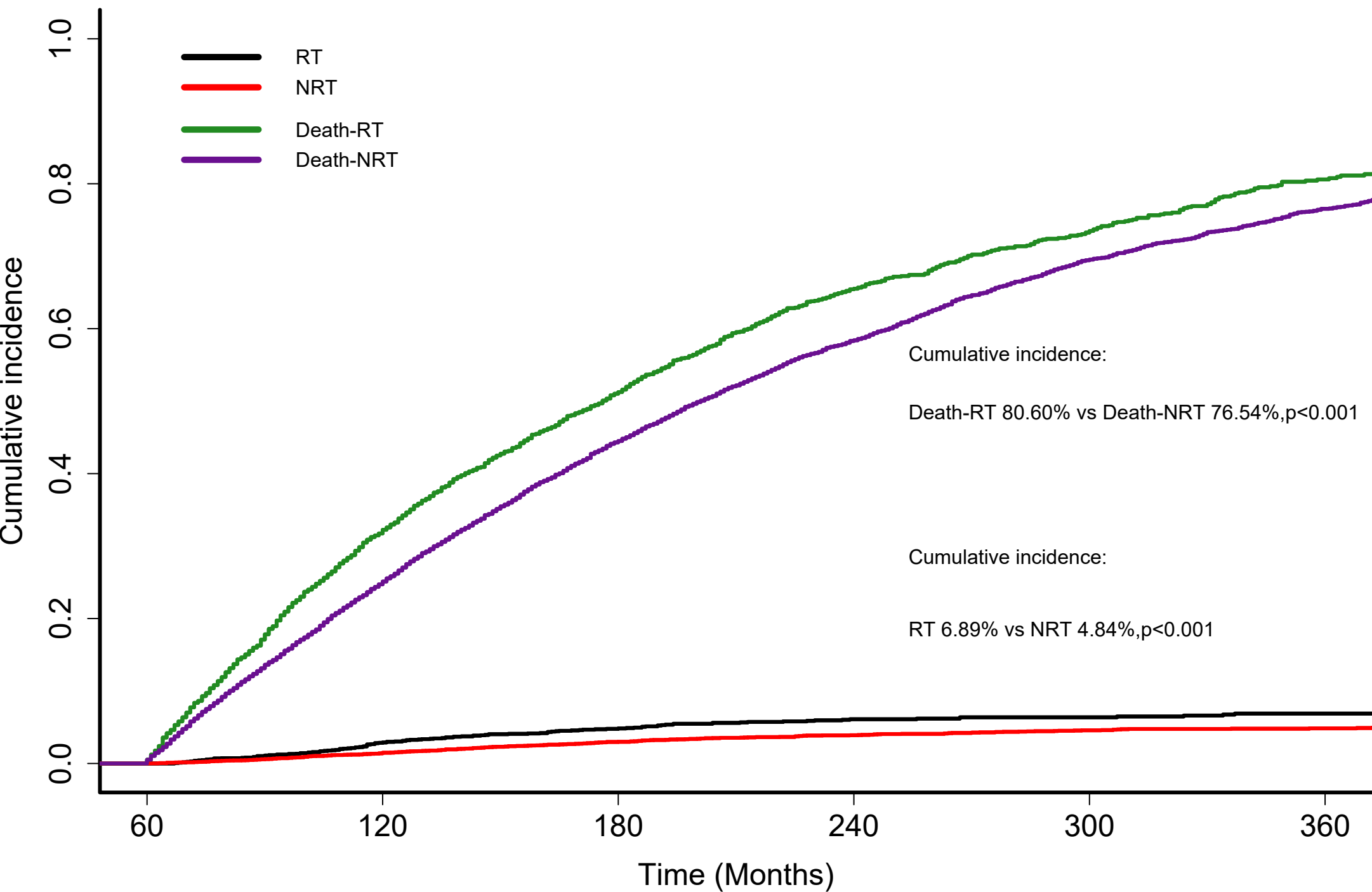

Supplement: Supplementary Figure S2 — Cumulative incidence and cumulative mortality of SLC between patients who received RT and those who did not receive RT. [file Data_Sheet_2.PDF]

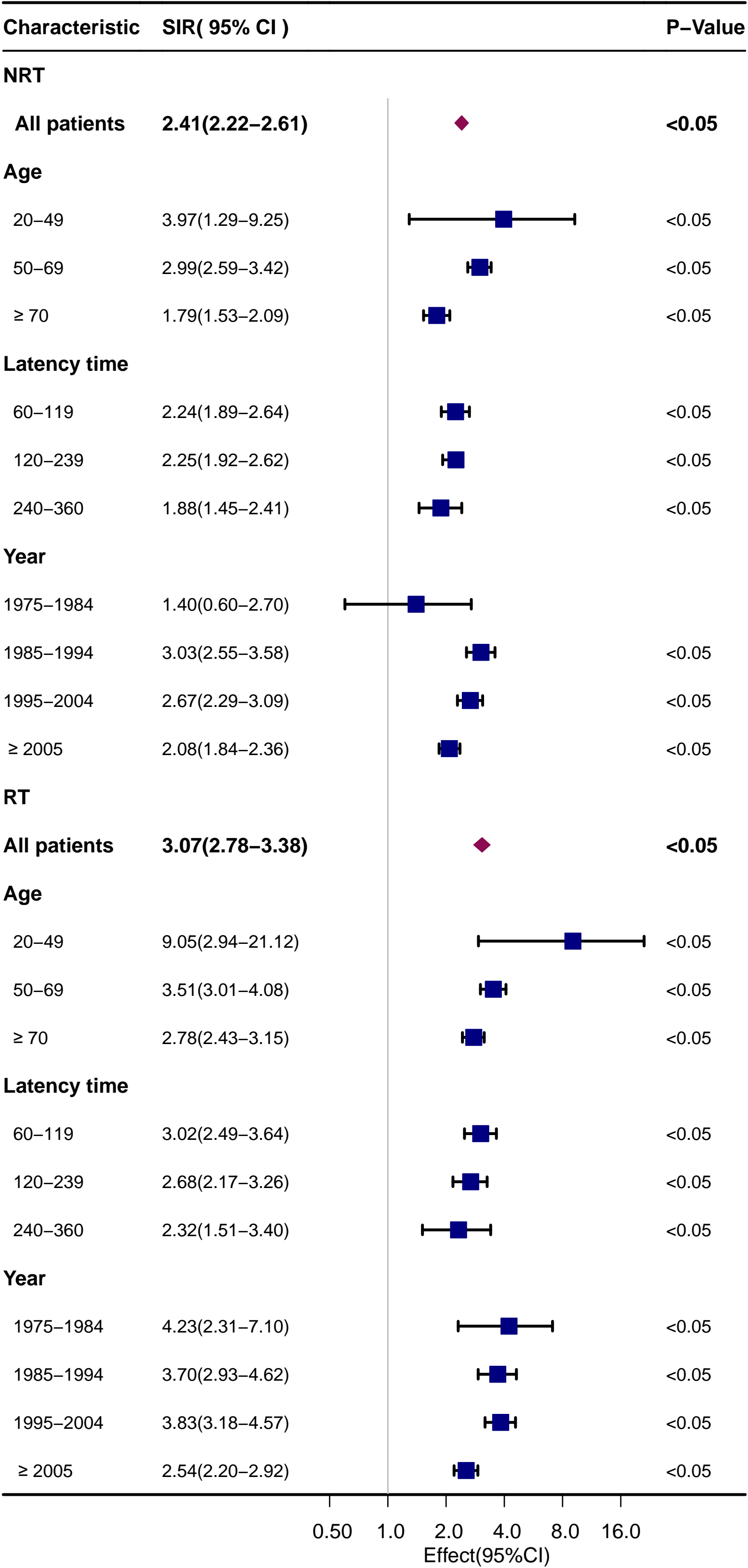

Supplement: Supplementary Figure S3 — SIR for SLC by age at diagnosis, latency, and year of OCC diagnosis. [file Data_Sheet_3.PDF]
