# Supplementary material for: Association between radiotherapy for surgically treated oral cavity cancer and secondary lung cancer
Source: Front Public Health. 2023 Mar 22;11:1120671. doi: 10.3389/fpubh.2023.1120671 (PMC10073750; doi:10.3389/fpubh.2023.1120671)
Supplement: Supplementary Table S1 — Poisson regression model for developing SLC in OCC. [file Table_1.pdf]

Table S1 Poisson regression model for developing SLC in OCC.

Variables	Unjusted RR (95% CI)	P	Adjusted RR (95% CI)	P
Age				
20-49	1 (Ref)		1(Ref)	
50-69	2.21(1.76-2.82)	<0.001	1.78 (1.41-2.29)	<0.001
≥ 70	1.00(0.74-1.36)	0.983	0.94 (0.68-1.28)	0.724
Sex				
Female	1 (Ref)		1(Ref)	
Male	1.46(1.23-1.73)	<0.001	1.26(1.06-1.50)	0.030
Race				
White	1 (Ref)		1(Ref)	
Black	1.16(0.85-1.55)	0.418	1.06(0.77-1.43)	0.751
Other[a]	0.50(0.31-0.75)	0.009	0.71(0.45-1.07)	0.197
Year				
1975-1984	1 (Ref)		1(Ref)	
1985-1994	0.88(0.72-1.06)	0.253	0.97(0.80-1.18)	0.829
1995-2004	0.61(0.49-0.75)	<0.001	0.74(0.60-0.92)	0.022
≥ 2005	0.16(0.11-0.22)	<0.001	0.20(0.14-0.28)	<0.001
Marital status				
Single	1 (Ref)		1(Ref)	
Married	1.42(1.09-1.89)	0.035	1.21(0.92-1.61)	0.269
Other/unknown[b]	1.47(1.11-1.99)	0.029	1.35(1.01-1.84)	0.097
Site				
Lip	1(Ref)		1(Ref)	
Tongue	0.70(0.50-1.01)	0.098	0.78(0.55-1.12)	0.250
Gum	0.87(0.58-1.31)	0.583	0.96(0.63-1.46)	0.866
Floor of Mouth	2.00(1.47-2.78)	<0.001	1.57(1.14-2.21)	0.025
Palate	0.56(0.34-0.91)	0.053	0.93(0.54-1.55)	0.814
Other	1.30(0.93-1.86)	0.213	1.37(0.96-1.99)	0.154
Grade				
Grade I/II	1 (Ref)		NA	
Grade III/IV	1.11(0.84-1.44)	0.527	NA	NA
Unknown	1.10(0.90-1.32)	0.421	NA	NA
Histology				
Squamous cell carcinoma	1 (Ref)		1(Ref)	
Other	0.46(0.35-0.59)	<0.001	0.55(0.40-0.74)	0.001
Stage				
Localized	1 (Ref)		1(Ref)	
Regional	1.34(1.14-1.57)	0.003	1.00(0.83-1.20)	0.984
Chemotherapy				
No	1 (Ref)		1(Ref)	
Yes	0.99(0.65-1.44)	0.969	1.13(0.73-1.67)	0.637

Radiotherapy				
No	1 (Ref)		1(Ref)	
Yes	1.41（1.19-1.67）	0.001	1.33(1.09-1.60）	0.015

Abbreviations: HNM, Head and neck malignancy; SLC, Second primary lung cancer; OCC: oral cavity cancer; NA, Not available; RR, Relative risk; CI, Confidence interval. Reference, Ref.
Note: [a] Other including American Indian/AK Native, Asian/Pacific Islander. [b] Other including Divorced, Separated, Widowed, Unmarried or Domestic partner.
