# Supplementary material for: Association between radiotherapy for surgically treated oral cavity cancer and secondary lung cancer
Source: Front Public Health. 2023 Mar 22;11:1120671. doi: 10.3389/fpubh.2023.1120671 (PMC10073750; doi:10.3389/fpubh.2023.1120671)
Supplement: Supplementary Table S2 — Subgroup analyses of competing risk regression for the risk of developing SLC. [file Table_2.pdf]

Table S2. Subgroup analyses of competing risk regression for the risk of developing SLC.

Subgroup	RT (events/total)	NRT(events/total)	HR (95% CI)	P-Value
Age (years)				
20-49	14/602	43/1816	NA	NA
50-69	103/1586	209/4397	1.37(1.09-1.71)	0.023
≥ 70	19/522	41/2013	NA	NA
Sex				
Female	49/1084	95/3555	1.51(1.09-2.11)	0.040
Male	87/1626	198/4671	1.29(1.01-1.63)	0.091
Race				
White	118/2266	264/7239	1.40(1.13-1.73)	0.009
Black	13/236	19/450	NA	NA
Other[a]	5/208	10/537	NA	NA
Year				
1975-1984	40/518	108/1911	1.24(0.90-1.70)	0.270
1985-1994	45/666	101/2072	1.23(0.90-1.70)	0.280
1995-2004	35/751	72/2127	1.47(0.94-2.31)	0.160
≥ 2005	16/775	12/2116	NA	NA
Marital status				
Single	10/397	32/1079	0.61(0.29-1.27)	0.270
Married	82/1584	177/4822	1.43(1.12-1.81)	0.015
Other/unknown[b]	44/729	84/2325	1.55(1.06-2.25)	0.058
Site				
Lip	1/41	30/878	NA	NA
Tongue	26/778	52/2507	1.46(0.92-2.32)	0.180
Gum	10/346	25/842	0.86(0.41-1.82)	0.750
Floor of Mouth	60/773	127/2002	1.27(0.96-1.70)	0.160
Palate	4/196	14/751	NA	NA
Other	35/576	45/1246	1.81(1.22-2.70)	0.014
Grade				
Grade I/II	96/1805	189/5691	1.41(1.11-1.78)	0.016
Grade III/IV	16/448	26/547	0.97(0.52-1.80)	0.940
Unknown	24/457	78/1988	1.39(0.89-2.17)	0.290
Histology				
Squamous cell carcinoma	127/2293	260/6539	1.40(1.14-1.71)	0.007
Other	9/417	33/1687	NA	NA
Stage				
Localized	51/938	209/6432	1.61(1.23-2.08)	0.003
Regional	85/1772	84/1803	1.13(0.86-1.47)	0.470
Chemotherapy				
No	118/2297	293/8176	1.29(1.06-1.58)	0.035
Yes	18/413	0/50	NA	NA

Note: NA, not applicable; NRT, no radiation therapy; RT, radiation therapy.

Note: [a] Other including American Indian/AK Native, Asian/Pacific Islander. [b] Other including Divorced, Separated, Widowed, Unmarried or Domestic partner.
