# Supplementary material for: Association between radiotherapy for surgically treated oral cavity cancer and secondary lung cancer
Source: Front Public Health. 2023 Mar 22;11:1120671. doi: 10.3389/fpubh.2023.1120671 (PMC10073750; doi:10.3389/fpubh.2023.1120671)
Supplement: Supplementary Table S3 — Baseline characteristics of patients with SLC in the original/matched data set. [file Table_3.pdf]

Table S3 Baseline characteristics of patients with SLC in the original/matched data set.

Variables	Original data set			Matched data set		
	NRT (n = 293)	RT (n = 136)	p	NRT (n = 134)	RT (n = 134)	p
Age, n (%),years			0.143			0.903
20-49	5 (1.7)	0 (0)		0 (0.0)	0 (0.0)	
50-69	116 (39.6)	64 (47.1)		63 (47.0)	64 (47.8)	
≥ 70	172 (58.7)	72 (52.9)		71 (53.0)	70 (52.2)	
Sex, n (%)			0.462			0.519
Female	95 (32.4)	49 (36.0)		43 (32.1)	48 (35.8)	
Male	198 (67.6)	87 (64.0)		91 (67.9)	86 (64.2)	
Race, n (%)			0.506			0.312
White	264 (90.1)	118 (86.8)		123 (91.8)	116 (86.6)	
Black	19 (6.5)	13 (9.6)		9 (6.7)	13 (9.7)	
Other/unknown[a]	10 (3.4)	5 (3.7)		2 (1.5)	5 (3.7)	
Year, n (%)			0.769			0.958
1975-1984	7 (2.4)	3 (2.2)		3 (2.2)	3 (2.2)	
1985-1994	82 (28.0)	32 (23.5)		33 (24.6)	32 (23.9)	
1995-2004	108 (36.9)	49 (36.0)		51 (38.1)	48 (35.8)	
≥2005	96 (32.7)	52 (38.3)		47 (35.1)	51 (38.1)	
Marital status, n (%)			0.105			0.455
Single	37 (12.6)	8 (5.9)		5 (3.7)	8 (6.0)	
Married	148 (50.5)	74 (54.4)		82 (61.2)	73 (54.5)	
Other/unknown[b]	108 (36.9)	54 (39.7)		47 (35.1)	53 (39.6)	
Anatomic sites, n (%)			0.044			0.907
Main bronchus	12 (4.1)	11 (8.1)		8 (6.0)	11 (8.2)	
Upper lobe	138 (47.1)	70 (51.5)		74 (55.2)	69 (51.5)	
Middle lob	16 (5.5)	11 (8.1)		12 (9.0)	10 (7.5)	
Lower lob	73 (24.9)	32 (23.5)		29 (21.6)	32 (23.8)	
Unknown	54 (18.4)	12 (8.8)		11 (8.2)	12 (9.0)	
Grade, n (%)			0.013			0.934
Grade I/II	69 (23.5)	21 (15.4)		19 (14.2)	21 (15.7)	
Grade III/IV	89 (30.4)	60 (44.1)		58 (43.3)	58 (43.3)	
Other/Unknown	135 (46.1)	55 (40.5)		57 (42.5)	55 (41.0)	
Histology, n (%)			0.229			0.463
Small cell carcinoma	45 (15.4)	15 (11.0)		19 (14.2)	15 (11.2)	
Non-small cell carcinoma	248 (84.6)	121 (89.0)		115 (85.8)	119 (88.8)	
Stage, n (%)			0.773			0.888
Localized	57 (19.5)	31 (22.8)		29 (21.6)	31 (23.1)	
Regional	76 (25.9)	35 (25.7)		34 (25.4)	34 (25.4)	
Distant	124 (42.3)	57 (41.9)		54 (40.3)	56 (41.8)	
Unknown	36 (12.3)	13 (9.6)		17 (12.7)	13 (9.7)	
Surgery			0.863			1.000

No	222 (75.8)	102 (75.0)		100 (74.6)	100 (74.6)	
Yes	71 (24.2)	34 (25.0)		34 (25.4)	34 (25.4)	
Radiation, n (%)			0.747			0.615
No	174 (59.4)	83 (61.0)		85 (63.4)	81 (60.4)	
Yes	119 (40.6)	53 (39.0)		49 (36.6)	53 (39.6)	
Chemotherapy, n (%)			0.905			0.788
No	203 (69.3)	95 (69.9)		96 (71.6)	94 (70.1)	
Yes	90 (30.7)	41 (30.1)		38 (28.4)	40 (29.9)	

Abbreviations: HNM, Head and neck malignancy; PLC, Primary lung cancer; SLC, Second primary lung cancer; RT, Radiotherapy; NRT, No radiotherapy.

Note: [a] Other including American Indian/AK Native, Asian/Pacific Islander. [b] Other including Divorced, Separated, Widowed, Unmarried or Domestic partner.
