# Supplementary material for: Association between radiotherapy for surgically treated oral cavity cancer and secondary lung cancer
Source: Front Public Health. 2023 Mar 22;11:1120671. doi: 10.3389/fpubh.2023.1120671 (PMC10073750; doi:10.3389/fpubh.2023.1120671)
Supplement: Supplementary Table S4 — Baseline characteristics of patients with SLC and matched PLC in OCC. [file Table_4.pdf]

Table 4S Baseline characteristics of patients with SLC and matched PLC in OCC.

Variables	PLC-RT	SLC-RT		PLC-NRT	SLC-NRT	
	(n = 136)	(n = 136)	p	(n = 293)	(n = 293)	p
Age, n (%),years			0.011			0.138
20-49	0 (0)	0 (0)		5 (1.7)	13 (4.4)	
50-69	64 (47.1)	85 (62.5)		116 (39.6)	119 (40.7)	
≥70	72 (52.9)	51 (37.5)		172 (58.7)	161 (54.9)	
Sex, n (%)			0.195			0.007
Female	49 (36.0)	39 (28.7)		95 (32.4)	66 (22.5)	
Male	87 (64.0)	97 (71.3)		198 (67.6)	227 (77.5)	
Race, n (%)			0.392			0.659
White	118 (86.7)	115 (84.5)		264 (90.1)	268 (91.6)	
Black	13 (9.6)	11 (8.1)		19 (6.5)	14 (4.8)	
Other/unknown[a]	5 (3.7)	10 (7.4)		10 (3.4)	11 (3.8)	
Year, n (%)			0.001			0.805
1975-1984	3 (2.2)	1 (0.7)		7 (2.4)	3 (1.0)	
1985-1994	32 (23.5)	89 (65.4)		82 (28/0)	223 (76.1)	
1995-2004	49 (36.0)	46 (33.9)		108 (36.9)	67 (22.9)	
≥2005	52 (38.3)	0 (0)		96 (32.7)	0 (0)	
Marital status, n (%)			0.323			0.001
Single	8 (5.9)	8 (5.9)		37 (12.6)	67 (22.9)	
Married	74 (54.4)	62 (45.6)		148 (50.5)	109 (37.2)	
Other/unknown[b]	54 (39.7)	66 (48.5)		108 (36.9)	117 (39.9)	
Anatomic sites, n (%)			0.210			0.347
Main bronchus	11 (8.1)	6 (4.4)		12 (4.1)	6 (2.0)	
Upper lobe	70 (51.5)	76 (55.9)		138 (47.1)	144 (49.1)	
Middle lob	11 (8.1)	17 (12.5)		16 (5.5)	22 (7.5)	
Lower lob	32 (23.5)	21 (15.4)		73 (24.9)	61 (20.8)	
Unknown	12 (8.8)	16 (11.8)		54 (18.4)	60 (20.6)	
Grade, n (%)			0.134			0.091
Grade I/II	21 (15.4)	13 (9.6)		69 (23.5)	71 (24.2)	
Grade III/IV	60 (44.2)	75 (55.1)		89 (30.4)	111 (37.9)	
Other/Unknown	55 (40.4)	48 (35.3)		135 (46.1)	111 (37.9)	
Histology, n (%)			0.543			0.376
Small cell carcinoma	15 (11.0)	12 (8.8)		45 (15.4)	53 (18.1)	
Non-small cell carcinoma	121 (89/0)	124 (91.2)		248 (84.6)	240 (81.9)	
Stage, n (%)			0.001			0.001
Localized	31 (22.8)	24 (17.6)		57 (19.5)	37 (12.6)	
Regional	35 (25.7)	27 (19.9)		76 (25.9)	46 (15.7)	
Distant	57 (41.9)	40 (29.4)		124 (42.3)	68 (23.2)	
Unknown	13 (9.6)	45 (33.1)		36 (12.3)	142 (48.5)	
Surgery			0.781			0.507
No	102 (75.0)	100 (73.5)		222 (75.8)	215 (73.4)	

Yes	34 (25.0)	36 (26.5)		71 (24.2)	78 (26.6)	
Radiation, n (%)			0.220			0.278
No	83 (61.0)	73 (53.7)		174 (59.4)	161 (54.9)	
Yes	53 (39.0)	63 (46.3)		119 (40.6)	132 (45.1)	
Chemotherapy, n (%)			0.218			0.015
No	95 (69.9)	104 (76.5)		203 (69.3)	229 (78.2)	
Yes	41 (30.1)	32 (23.5)		90 (30.7)	64 (21.8)	

Abbreviations: HNM, Head and neck malignancy; PLC, Primary lung cancer; SLC, Second primary lung cancer; RT, Radiotherapy; NRT, No radiotherapy; OCC: Oral cavity cancer.

Note: [a] Other including American Indian/AK Native, Asian/Pacific Islander. [b] Other including Divorced, Separated, Widowed, Unmarried or Domestic partner.
